# Limited National Potential for Reducing Anesthesia Clinician Staffing for Cataract Surgery: A Historical Cohort Study Using a Large United States Anesthesia Registry

**DOI:** 10.7759/cureus.89895

**Published:** 2025-08-12

**Authors:** Richard H Epstein, Franklin Dexter, Steven Gayer, Richard P Dutton

**Affiliations:** 1 Anesthesiology, University of Miami Miller School of Medicine, Miami, USA; 2 Anesthesia, University of Iowa, Iowa City, USA; 3 Anesthesiology, United States Anesthesia Partners, Largo, USA

**Keywords:** anesthesiologists, cataract, ophthalmologists, workforce, workload

## Abstract

Background

Cataract surgery, the most frequently performed surgical procedure worldwide, is increasing, with many patients receiving intravenous sedation by anesthesia clinicians. Given anesthesia clinician shortages and ambulatory surgery facility scheduling constraints, some ophthalmologists are moving to office-based care without anesthesia services. We examined the potential impact of such a shift in care on reducing anesthesia clinician staffing.

Methods

We analyzed 622,953 cataract surgeries performed at 672 facilities between 2022 and 2023, using data from the American Society of Anesthesiologists’ national anesthesia registry, to determine the proportion of shortest lists that exceeded four hours on regular workdays. The number of anesthesia clinicians staffing these cases, by facility, was determined from the counts of cases with overlapping care during each workday minute. The time for the number of simultaneously running operating rooms (ORs) to decrease permanently by one on a specified day corresponds to the completion of the facility's shortest list.

Results

There was at least one cataract case for 80.3% (95% CI 79.4% to 81.3%) of all combinations of facilities and workdays. Among all facilities, the proportion of time for the OR to complete its shortest list exceeded four hours for 84.3% of combinations of quarter of the year and day of the week (95% CI, 81.8% to 86.8%).

Conclusions

The complete removal of anesthesia clinician staffing would likely result in a substantive disruption to the cataract surgery workload, given the number of patients receiving care during half-day sessions and the probability that some patients will require an anesthesia clinician to manage their intraoperative care.

## Introduction

Cataract surgery is the most common surgical procedure worldwide, and its incidence is increasing as the population ages [1]. Cataract surgery is considered a low-risk procedure [2,3] that does not routinely require multidisciplinary coordination or preoperative medical screening prior to the day of surgery [4]. The procedure is often performed using topical anesthetics, combined topical and intracameral anesthesia, sub-Tenon's injection, or sharp needle-based block [5]. A portion of these cases, performed in office-based settings, is accomplished with oral rather than intravenous sedation [6], although there is controversy about the safety of operating in such venues [7,8]. However, the involvement of an anesthesia clinician to provide sedation and monitoring is common. Uniformly, such clinicians include anesthesiologists, but depending on the country and permissible scope of practice, the anesthesia care team may also comprise certified registered nurse anesthetists, certified anesthesiology assistants, anesthesiology residents and fellows, and other trained healthcare professionals working under the direct supervision of an anesthesiologist. For example, in 2022, only 10% to 12% of United States Medicare beneficiaries who had cataract procedures did so without anesthesia services [3]. In Canada, there is wide variability among the provinces as to whether an anesthesia clinician is involved in the care of cataract patients, which appears to be driven in large part by reimbursement issues (Lecture: Aucoin S. Talk Shifting and Take Sharing in Evolving Canadian Anesthesiology Care Models. Canadian Anesthesiologists' Society Annual Meeting; June 22, 2025). The Ophthalmology Get it Right the First Time (GIRFT) Programme National Specialty Report underscored the need to address rising demand while maintaining patient safety and surgical outcomes [9].

There is a worldwide shortage of anesthesia clinicians, both in developing [10] and highly developed countries such as the United States [11] and the United Kingdom [9], which may contribute to reduced access to surgical care in the face of increasing demand. Decreasing the involvement of an anesthesia clinician in selected groups of patients undergoing cataract surgery who have very low perioperative complication rates may be rational from a public health perspective, as it may make more anesthesia clinicians available for the care of patients undergoing more physiologically complex surgeries.

The potential magnitude of the reduction in staffing and strategies to achieve such a reduction depends on the duration of the cataract surgical lists and the number of operating rooms (ORs) provided for cataract surgery by facility and day of the week. We use the term "list" to refer to the sequential group of patients having cataract surgery in an OR on a given date. Suppose it were the situation that many facilities are opening cataract surgery ORs for short lists of cases (i.e., only a few hours). If so, there may be opportunities to adjust the staffing to reduce the number of required anesthesia clinicians by reducing the number of planned sessions, for example, by consolidating lists from several days to one day. Further, if it is possible to construct daily lists consisting only of low-risk cataract procedures in patients with acceptable risk factors, suitable for topical anesthesia and light (oral) sedation only [12], it might be possible to reassign anesthesia clinicians previously providing such intraoperative care to other work.

Contrariwise, if most of the lists are long (e.g., at least 4.0 hours), there would be less opportunity to reduce staffing because of the greater probability that some patients might require anesthesia care due to substantive comorbidities, anxiety, or other conditions requiring a greater depth of sedation and more monitoring than non-anesthesia personnel might be able to manage. A recent study of patient perspectives regarding cataract surgery revealed a strong preference for anesthesia-led sedation, but with openness for alternative care if demonstrated to be safe [13]. In the most recent Ophthalmology GIRFT report, the expected OR throughput for cataract surgery was 30 minutes, with an expectation that eight patients would receive such care in a four-hour list [9]. Achieving such a goal "…often requires staff to facilitate faster turnaround and does not apply to more complex cases" [9]. Our objective was to identify the "recruitable workforce" that could potentially be reassigned to other work if they were not needed for low-risk cataract surgery.

We analyzed all cataract surgery procedures involving anesthesia services from United States healthcare facilities that contribute outcomes data to the American Society of Anesthesiologists (ASA) National Anesthesia Clinical Outcomes Registry [14]. For our primary analyses, we estimated the proportion of such facilities, by day of the week, that exceeded a four-hour (half-day) threshold among the shortest list of cataract surgical procedures performed at each facility. The United States is a suitable country for such analysis because approximately 90% of all surgery is provided on an ambulatory basis [15]. Furthermore, many facilities open more ORs at the start of the day than are justified by considerations of maximizing OR efficiency [16-18]. Such behavior is in line with patient preferences for having morning rather than afternoon cataract surgery [19].

## Materials and methods

This study was performed following the execution of a data use agreement with the ASA's Anesthesia Quality Institute to use de-identified National Anesthesia Clinical Outcomes Registry (NACOR) data [14]. The University of Miami Institutional Review Board determined that this retrospective study of de-identified data constituted non-human subjects research; thus, patient consent was not required. The Strengthening the Reporting of Observational Studies in Epidemiology (STROBE) checklist for observational studies was followed.

Dataset description

**Figure 1:**
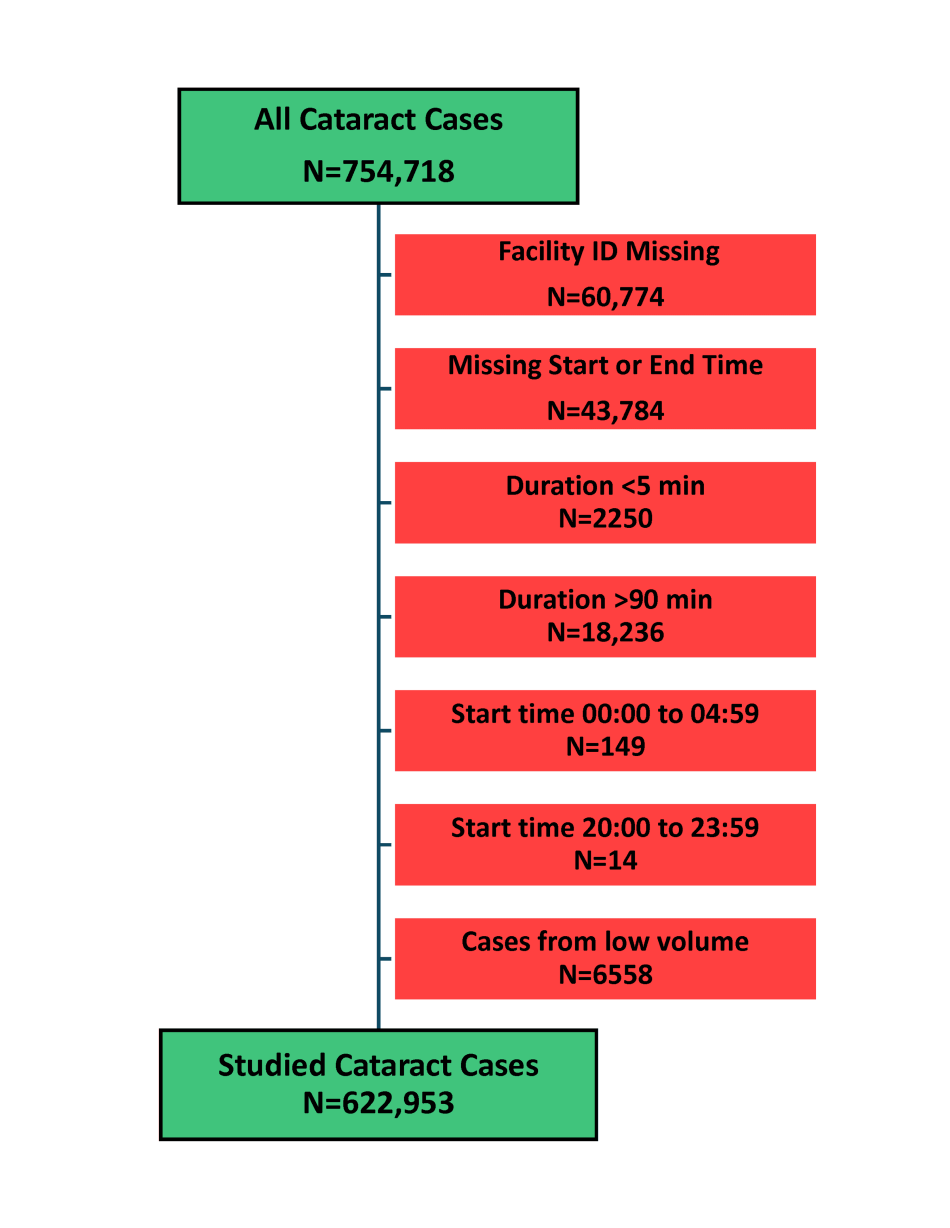
Flow diagram to identify elective cataract cases Cases with implausible durations for primary elective cataract surgery were excluded. The five-minute threshold corresponds to the 0.01 percentile, and the 90-minute threshold to the 0.9725 percentile. Cases starting during late evening and early morning hours were also excluded, as they likely represent either portions of urgent or emergent surgery or miscoding, given that elective cataract surgeries would be expected to start rarely after 8 PM or before 5 AM. Of the 754,718 cataracts identified in the dataset, 131,765 (17.5%) were excluded.

The data analyzed included all cataract surgery cases performed from January 1, 2022, to December 31, 2023. Cataract surgery was identified by the presence of the Current Procedure Terminology [20] anesthesiology code 00142 (anesthesia for procedures on the eye; lens surgery) or Clinical Classifications Software procedure code 15 (lens and cataract procedures) [21]. Among the 754,718 cases identified, 622,953 were included after applying the exclusion criteria (Figure 1). For each studied case, data elements included the facility identifier, the procedure date, the day of the week, and the anesthesia start and end times. The year and quarter of the procedure date were calculated, and the start and end times were converted to the offset, in minutes, from midnight (e.g., 7:00 AM = 420 minutes) to facilitate the identification of overlapping ORs.

Determination of the number of ORs running simultaneously

**Figure 2:**
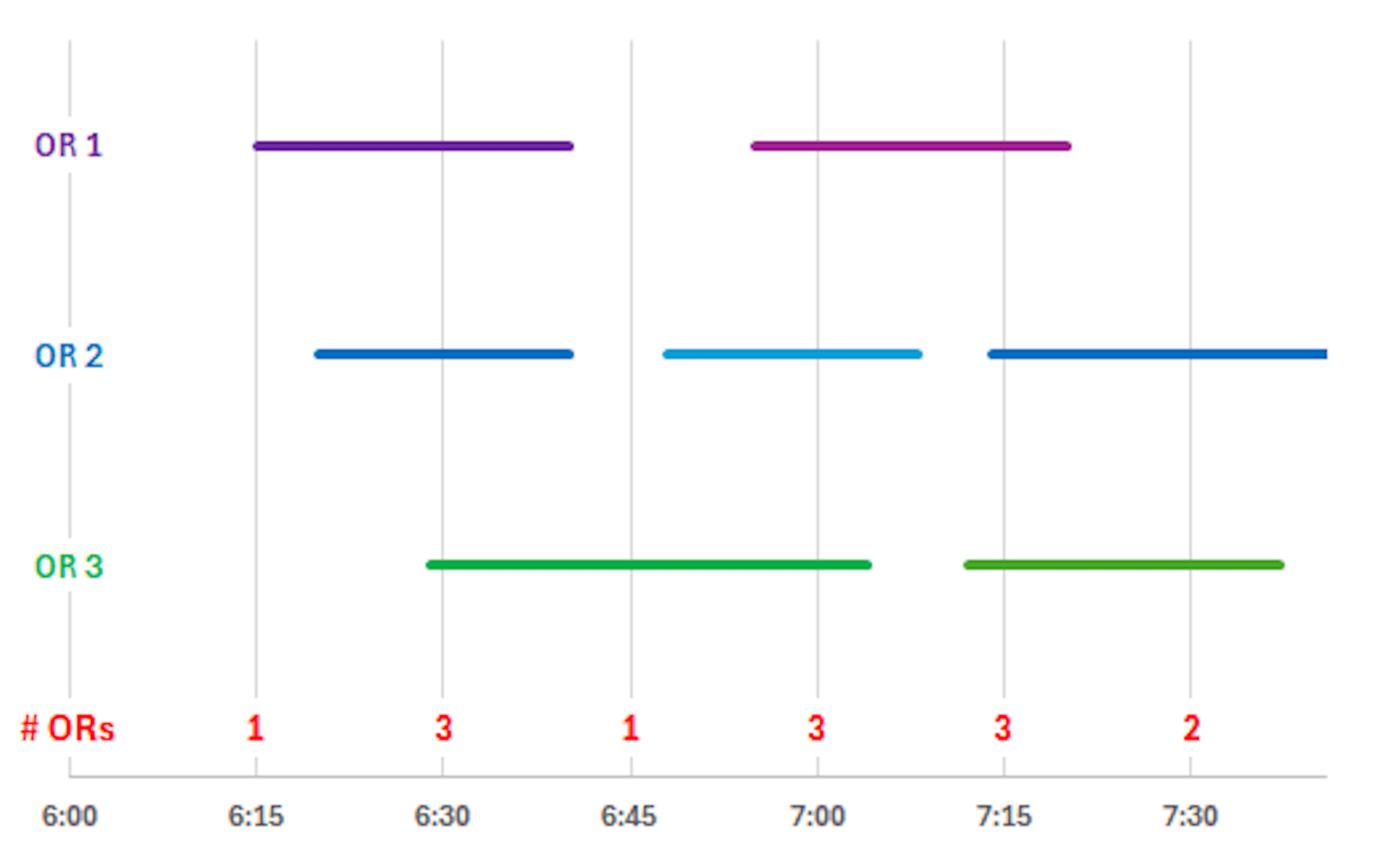
Determination of the number of simultaneously running ORs (i.e., number of anesthesia clinicians providing care) The number of simultaneously running cases (horizontal bars), equivalent to the number of anesthesia clinicians providing care, was inferred from the number of overlapping cases in each 1-minute bin of the day (red numbers at the bottom of the graph). The effective start of the workday was the first time the maximum number of simultaneous running ORs occurred, 6:30 AM in the example. That value corresponds to the number of anesthesia clinicians needed to staff the ORs performing cataract surgery. The process continued throughout the workday until 3:00 PM. The time until the OR with the shortest list ended corresponds to the first time when the number of ORs simultaneously running dropped by one and did not increase for the remainder of the workday.

The day was divided into 1-minute bins from 6:30 AM to 03:00 PM (390 minutes to 900 minutes from midnight), and for each case, the bin's value was set to 1 if the case was running during that bin and to 0 otherwise. Then, for each combination of facility and date, the total number of ORs in which cataract cases were running simultaneously during each bin was determined by summing the bins. The algorithm for determining the number of cases running is illustrated graphically in Figure 2.

Because a given anesthesia clinician can only provide direct patient care in one OR during any given minute of the day, the number of simultaneously running ORs is equal to the number of anesthesia clinicians providing such anesthesia care during that interval. This calculation of clinicians does not include the anesthesiologists who are supervising multiple ORs (not provided in the database), which will vary depending on the supervision ratio. The number of ORs opened at the start of each date at each facility was determined as the maximum number of simultaneously running ORs between 6:30 AM and 8:29 AM. While some facilities started a few ORs as early as 5:45 AM on some dates, the maximum number of ORs running occurred after 6:30 AM. Thus, to calculate the effective start of each workday, the time at each facility on each date when the maximum number of ORs was first running was used. The durations of the lists were then determined as the difference in minutes from the end time of the cases and the effective start of the workday. This process enabled the normalization of list durations across facilities that started work at different times on various days of the week, performed separately for each facility.

Determination of the proportion of workdays with at least one cataract case

Pooling all facilities, we calculated the proportion of regular workdays (Mondays through Fridays, excluding federal holidays) with at least one cataract case. To do so, for each combination of facility, quarter of the year, and day of the week, the number of regular workdays with at least one cataract case was measured for use as a numerator. For each of the n=9 quarters and n=5 weekdays, the maximum number of such regular workdays among all facilities was measured as a denominator. These two values, the numerator and the denominator, were summed among all facilities for each of the 40 combinations of quarter and weekday. The ratio of the sums among these N=40 numerators and denominators was the point estimate for the proportion of workdays with at least one cataract case. The standard error was calculated using jackknife estimates of the ratio of the sums, including their covariances, among the N=40 combinations. The 95% confidence interval was then calculated using Student's t-distribution for one group.

Determination of the time until the number of simultaneously running ORs was reduced by one (i.e., the shortest list completed)

For each facility and date, we determined the interval from the effective start of the workday until the first time when the count of simultaneously running ORs decreased by one and did not increase over the remainder of the workday. This specification ensured that the end of the day was not falsely noted due to a transient decrease in the OR count by minute due to the flux of patients entering and leaving the rooms. These intervals correspond to the time until the OR with the shortest list of cataract cases was completed. If that decrease did not occur by 3:00 PM, then the interval was set to the difference between the effective start of the workday and 3:00 PM. For each combination of facility, year, quarter, and day of the week, the number of days on which cataract surgery was performed was determined. Only combinations with at least six operating days in the year and quarter were included for subsequent analysis of the percentiles. This limitation was applied because there are 13 combinations for each weekday among the 13 weeks in each quarter, and we needed to exclude facilities that did not run cataract surgery lists regularly on that day of the week to avoid biasing the results.

Calculation of the optimal order quantity of OR time for the least busy anesthesia clinician

For each combination of facility, quarter, and regular day of the week, we calculated the optimal order quantity of OR time for the least busy anesthesia clinicians caring for cataract surgery patients. In the context of this study, the term "optimal order quantity" refers to the ideal amount of OR time that should be provided to a surgical service to minimize the weighted combination of the costs of overutilized and underutilized OR time. This step is necessary because in the USA, there are generally not "sessions" into which cases are scheduled, and regardless, these are not reported to NACOR, only the cases performed.

Our methodology was based on the seminal paper published in 1997 by Strum et al., which demonstrated that the mathematics of the classical newsvendor problem can be applied to minimize the cost of using OR staff [22,23]. In this formulation, the analogy is to the optimal number of newspapers that the streetcorner newsvendor should purchase on each day of the week based on the historical number of newspapers sold on that day, the difference between the newsvendor's cost per newspaper and its sale price, and the loss of profit attributable to stocking out (i.e., demand but no more newspapers to sell). The optimal order quantity for a given OR is the number of hours that would need to be staffed (i.e., paid for) that minimizes the sum of the cost of underutilized time (i.e., paid hours - worked hours, when positive) plus the cost of overutilized time (i.e., worked hours - paid hours, when positive). To simplify the mathematics for decision-making, the cost of underutilized time is set to 1, and the cost of overutilized time is set to a multiple of 1. For example, the cost ratio of 2.0, used in our primary analyses, corresponds to a direct cost of 1.5 ("time-and-a-half" for overtime) plus 0.5 (for the intangible cost of recruitment and onboarding for staff who may leave because of forced, unwanted work beyond regular hours). If a suite had two ORs for cataract cases, both staffed for eight hours, and on a given day, the first was used for seven hours and the second for nine hours, the cost of the inefficiency of use of operating time for the two ORs on that day was the sum, 1 + 1 x 2 = 3 hours. This approach avoids including the actual cost per hour, as it cancels out, and one can simply relate the cost to the number of hours. The optimization problem involves calculating the number of staffed hours such that the sum of each day's cost over the range of dates results in the minimum value. The optimal order quantity in units of hours for each OR can be obtained by taking a percentile of the distribution of the daily hours of cases, as described in the next section.

Following Strum et al.'s methodology, we calculated the 66.7^th^ percentile, which corresponds to a cost ratio of 2.0, for our primary analysis. The 66.7^th^ percentile is calculated as $\tiny 100\times \frac{\text{2 hr overutilized}}{\text{2 hr overutilized}+\text{1 hr underutilized}}$, where the cost ratio of 2.0 refers to the ratio of "2 hr overutilized" to "1 hr underutilized."

We limited the calculation for the 66.7^th^ percentile to a sample size of ≥5 days to exclude at least one (i.e., the highest) ordered quantity, which could potentially be an outlier. To see that at least one quantity will be excluded when the 66.7^th^ percentile is calculated, consider a sample size of five. The position of the 66.7^th^ (i.e., 2/3rd) percentile value is given by the formula $\tiny \frac{2}{3}\left(5+1\right)=5$, which excludes the 5th ordered value. Had a sample size of four been selected, the largest ordered value would not have been excluded. The benefit of applying this inclusion criterion for determining the minimum number of historical cases needed to accurately calculate these critical percentiles was previously demonstrated by Wachtel and Dexter (2007) [24].

Determination of the percentiles of the intervals until the number of simultaneously running ORs was reduced by one (i.e., the shortest list completed)

For each combination of year and quarter, facility, and day of the week, we calculated the 66.7^th^ percentile among the pooled times until the number of simultaneously running ORs was reduced by one. This calculation was performed in SQL Server (Microsoft, Redmond, WA) using the percentile_cont function, which corresponds to the R quantile function, utilizing method 7 for linear interpolation (The R Foundation, Vienna, Austria) [25].

Analysis of potential staffing decisions to reduce the number of anesthesia clinicians providing care for cataract surgery patients by one

Statistical analyses were performed using Stata v19.5 (StataCorp, College Station, TX). We considered that facilities could make staffing decisions quarterly to reduce by one anesthesia clinician the number of staff who care for patients undergoing cataract surgery. These analyses were performed among all facilities on days when at least one OR was opened at the start of the day for cataract surgery and where at least two ORs were opened. For the first analysis, the reduction relates to completing the cataract surgery list when only one OR was opened for cataract surgery, or the shortest list when more than one OR was opened. The second analysis relates to completing the shortest list among two or more ORs. For example, if the facility opened three ORs, the determination would be for the time until only two ORs had cases running simultaneously. For each facility, we calculated a denominator based on the number of combinations of quarter and day of the week that each met the minimum sample size estimate for the number of days with at least one cataract case. For our primary analysis, we similarly calculated a numerator for the number of these combinations of quarter and day of the week for which the critical 66.7^th^ percentile exceeded four hours. We also used a four-hour threshold (half-day session) for the primary analysis because, given the short duration of cataract surgery, a substantive number of cases (approximately eight) would be expected to be completed during such an interval.

Analysis of the national impact of potential staffing decisions to reduce the number of anesthesia clinicians providing care for cataract surgery patients by one

**Figure 3:**
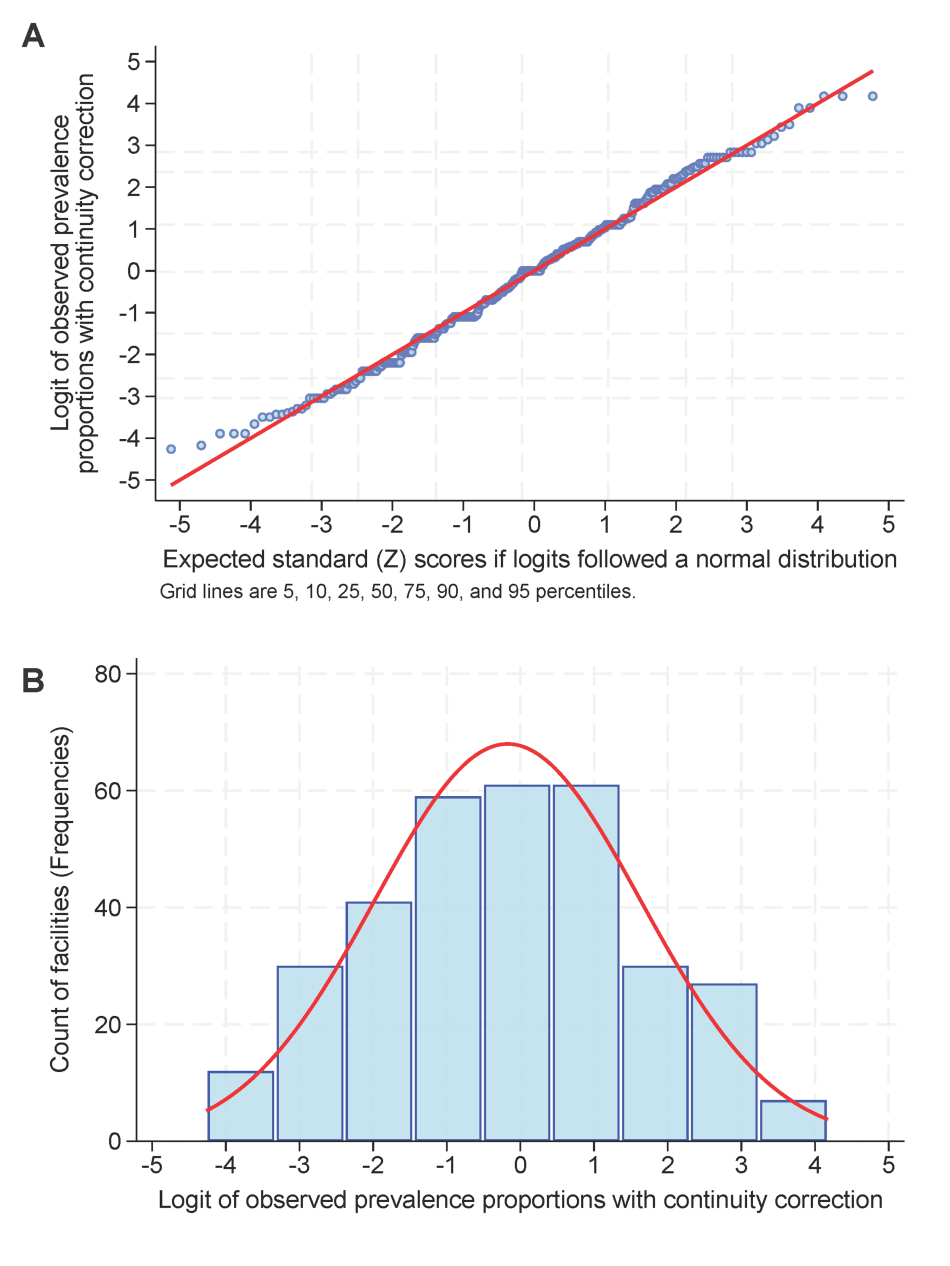
Distribution of the median proportions among facilities The median proportions for the 66.7^th^ percentile exceeding six hours were examined and confirmed to follow a normal distribution by the Shapiro-Wilk test (W=0.99, P=0.24). In the upper panel (A), the logits of the observed proportions closely follow the expected Z scores for a normal distribution. In the lower panel (B), the histogram of the logits closely follows the normal distribution (red line).

To summarize the results among facilities and infer the national impact, we used mixed-effects logistic regression with a random effect of facility to calculate marginal estimates of the proportions of quarter and day of the week combinations with the critical 66.7^th^ percentile exceeding four hours (Figure 3). The marginal estimates are the weighted mean proportions among facilities and are thus the relevant ones nationally. The marginal estimates' confidence intervals for each combination of critical percentile and minimum hours of interest were calculated using Student's t-distribution with degrees of freedom equal to the number of facilities for the combination minus one. Gaussian-Hermite quadrature was performed using 20 integration points. These calculations were performed using the *melogit *and *margins *commands. The marginal mean days with at least one cataract case, averaged over facilities, reported by the combination of quarter and day of the week, were similarly calculated using mixed-effects linear modelling.

Sensitivity analyses

We repeated the above calculations for conditional estimates. These are the median proportions among facilities. Ideally, the conditional estimates would be comparable to the marginal estimates.

We also performed the above calculations using cost ratios of 1.5 and 4.0, corresponding to the 60^th^ percentile ( $\tiny100\times \frac{1.5}{1.5+1.0}$) and 80^th^ percentile ( $\tiny100\times \frac{4.0}{4.0+1.0}$), respectively, of the distribution of the daily hours of cases. The minimum sample sizes for the 60^th^ and 80^th^ percentiles were four and nine historical days, respectively, following the formula described above to ensure exclusion of at least the highest ordered value. The series of cost ratios, ranging from 1.5 to 4.0, reflects an increasing aversion by the facility to working beyond regularly scheduled hours. For example, a cost ratio of 4.0 indicates that a facility values the cost of one hour of overtime the same as the cost of an OR ending four hours early.

As further sensitivity analyses, we considered six- and eight-hour thresholds for the critical percentile calculations. Values of four, six, and eight hours are typical choices for staff scheduling.

Statistical reporting of results

We report 95% confidence intervals without adjustment because the intervals are being used to show the precision of the estimates, some of which are quite wide. No statistical comparisons were made, and there were no minimally important managerial differences or equivalents. The data analysis and statistical plan were based on our previous research related to the use of the NACOR database [18] and an analysis of anesthesia clinician staffing based on overlapping cases [26]. A power analysis was not performed because all data were used (i.e., not a sample) and no statistical comparisons were made between groups.

## Results

There were 3127 facilities reporting cases to NACOR, among which 743 performed at least one cataract procedure. From the total population of 754,718 cases identified as involving cataract surgery, we studied 622,953 cases from 671 facilities after excluding cases with missing or implausible data and those from low-volume facilities (<104 cases in two years, Figure 1).

There was at least one cataract case for 80.3% (95% CI 79.4% to 81.3%) of all combinations of facilities and individual workdays. Facilities opening a single room for cataract surgery represented 71.3% ($\tiny 100\times \frac{328-94}{328}$) of the total.

Table 1 presents results for the proportions of quarter and weekday combinations for which the shortest list of cataract cases would have ideal budgeted staffing exceeding threshold hours among facilities performing cataract surgery. The critical percentile for the 66.7^th^ percentile (cost ratio 2.0) exceeded four hours for 84.3% of quarters and weekdays (95% CI 81.8% to 86.8%, Table 1). Note that the average facility has no quarter and weekdays for which the shortest-running OR with cataract cases would be planned for more than 8.0 hours (Table 1). In contrast, the average facility would have the large majority of its quarter and weekdays for which suitable staff scheduling would exceed 4.0 hours (Table 1). Therefore, ophthalmologists generally are doing substantive lists, rather than just a few cases.

**Table 1:** Mean marginal estimates and mean conditional estimates for the proportions of quarter and weekday combinations with different workdays for the least busy (fewest total hours) anesthesia clinician performing cataract surgery cases only that exceeded the threshold number of hours. The marginal and conditional estimates were calculated using a mixed-effects logistic regression model. ^a^ The 66.7^th^, 60^th^, and 80^th^ percentiles correspond to common cost ratios of overutilized to underutilized time of 2.0, 1.5, and 4.0, respectively. The primary analyses correspond to the mean marginal estimate and the mean conditional estimate for the 66.7^th^ percentile and a threshold of 4.0 hours. Secondary analyses are conducted for the six- and eight-hour thresholds at the 66.7^th^ percentile and for all threshold hours at the 60^th^ and 80^th^ percentiles. ^b^ Among facilities, the marginal prevalence proportion is the mean percentage of combinations of quarter and day of the week where the optimal number of allocated hours to minimize the sum of the cost of overutilized and underutilized time exceeded 4.0, 6.0, or 8.0 hours. The marginal mean is relevant nationally, being the weighted average among facilities of the proportions. ^c^ For each facility, we calculated the proportion of time periods (defined by quarter and day of the week) where the predicted optimal hours exceeded the listed threshold hours. The mean conditional estimate is the median of these proportions across all facilities. The mean conditional estimate is reported along with its 95% confidence interval. CI=confidence interval; IQR=interquartile range

Measurement	Threshold hours^a^ = 4.0	Threshold hours^a^= 6.0	Threshold hours^a^= 8.0
Mean marginal estimate^b^ (95% CI) for the prevalence proportion where the 66.7^th^ percentile > threshold hours	84.3% (81.8%–86.8%)	46.4% (42.7%–50.2%)	1.4% (0.5%–2.4%)
Mean conditional estimate^c^ (95% CI) for the prevalence proportion where the 66.7^th^ percentile > threshold hours	93.2% (91.0%–94.9%)	46.4% (42.7%–50.2%)	1.4% (0.5%–2.4%)
Mean marginal estimate^b^ (95% CI) for the prevalence proportion where the 60^th^ percentile > threshold hours	80.7% (78.0%–83.4%)	41.6% (38.0%–45.2%)	1.3% (0.1%–2.4%)
Mean conditional estimate^c^ (95% CI) for the prevalence proportion where the 60^th^ percentile > threshold hours	89.9% (87.3%–92.1%)	41.6% (38.0%–45.2%)	0.0% (0.0%–0.2%)
Mean marginal estimate^b^ (95% CI) for the prevalence proportion where the 80^th^ percentile > threshold hours	92.6% (90.4%–94.8%)	62.1% (57.7%–66.5%)	2.7% (1.4%–4.0%)
Mean conditional estimate^c^ (95% CI) for the prevalence proportion where the 80^th^ percentile > threshold hours	99.0% (98.0%–99.5%)	62.1% (57.7%–66.5%)	0.0% (0.0%–0.1%)

Table 2 presents the counts of days when cataract surgery was performed, quarter and day-of-the-week combinations, and the number of facilities included in the analyses of Table 1. The total number of cataract surgeries and the pooled mean number of cataract surgeries per day are also shown.

**Table 2:** Categorical counts corresponding to the analyses for the specified percentiles in Table 1. ^a^ The median (interquartile range) for the list duration was 6.03 (4.67–7.12) hours. ^b^ The median (interquartile range) for the list duration was 5.73 (4.41–6.95) hours. ^c^ The median (interquartile range) for the list duration was 6.77 (5.44–7.45) hours.

Category	Number corresponding to the prevalence proportions for the 66.7^th^ percentile^a^	Number corresponding to the prevalence proportions for the 60^th^ percentile^b^	Number corresponding to the prevalence proportions for the 80^th^ percentile^c^
Total days with cataract surgery	42,224	43,064	31,016
Quarter and day-of-the-week combinations	4516	4726	2829
Facilities	328	338	259
Total cataract surgeries	544,840	555,574	429,770
Mean cataract surgeries/day	12.9	12.9	13.9
Total cataract surgeries	544,840	555,574	429,770
Mean cataract surgeries per day	12.9	12.9	13.9

Among facilities opening at least two ORs at the start of the day for cataract surgery, the corresponding estimate was 63.0% of quarters and weekdays (95% CI 56.5% to 69.6%, Table 3). Facilities opening more than one OR for cataract surgery represented 28.7% (94/328) of the total (Tables 1, 2). Note that the average (median) facility has no quarter and weekdays for which the shortest-running OR with cataract cases would be planned for more than 8.0 hours (Table 2). In contrast, the average facility would have the large majority of its quarters and weekdays for which suitable staff scheduling would exceed 4.0 hours (Table 2). Therefore, even when considering the briefest of the cataract surgery ORs, ophthalmologists are doing substantive lists.

**Table 3:** Mean marginal estimates and mean conditional estimates for the proportions of quarter and weekday combinations with different workdays for the least busy (fewest total hours) anesthesia clinician performing cataract surgery cases only that exceeded the threshold number of hours, but only for days where at least two operating rooms were opened for such cases. The marginal and conditional estimates were calculated using a mixed-effects logistic regression model. No results are given for the threshold >8 hours because the observed proportions were 0.2% (2/940), 0.0% (0/1086), and 0.8% (4/506) for the 66.7^th^, 60^th^, and 80^th^ percentiles. ^a^ The 66.7^th^, 60^th^, and 80^th^ percentiles correspond to common cost ratios of overutilized to underutilized time of 2.0, 1.5, and 4.0, respectively. The primary analyses correspond to the mean marginal estimate and the median conditional estimate for the 66.7^th^ percentile and a threshold of 4.0 hours. Secondary analyses are for the threshold of 6.0 hours for the 66.7^th^ percentile and six and 8.0 hours for the 60^th^ and 80^th^ percentiles. ^b^ Among facilities, the marginal prevalence proportion is the mean percentage of combinations of quarter and day of the week where the conditional estimate for the optimal number of allocated hours to minimize the sum of the cost of overutilized and underutilized time exceeded 4.0 or 6.0 hours. The marginal mean is relevant nationally, being the weighted average among facilities of the proportions. ^c^ For each facility, we calculated the proportion of time periods (defined by quarter and day of the week) where the predicted optimal hours exceeded the listed threshold hours. The mean conditional estimate is the median of these proportions across all facilities. The mean conditional estimate is reported along with its 95% confidence interval. CI=confidence interval

‘Measurement	Threshold hours^a ^= 4.0	Threshold hours^a^= 6.0
Mean marginal estimate^b^ (95% CI) for the prevalence proportion where the 66.7^th^ percentile > threshold hours	63.0% (56.5%–69.6%)	29.3% (22.6%–36.0%)
Mean conditional estimate^c^ (95% CI) for the prevalence proportion where the 66.7^th^ percentile > threshold hours	68.0% (59.2%–75.7%)	18.1% (10.9%–28.6%)
Mean marginal estimate^b^ (95% CI) for the prevalence proportion where the 60^th^ percentile > threshold hours	57.6% (51.2%–63.9%)	24.1% (18.2%–30.0%)
Mean conditional estimate^c^ (95% CI) for the prevalence proportion where the 60^th^ percentile > threshold hours	60.9% (51.8%–69.3%)	12.1% (7.1%–20.0%)
Mean marginal estimate (95% CI) for the prevalence proportion^a^ where the 60^th^ percentile > threshold hours^b^	85.8% (78.7%–92.8%)	50.9% (40.6%–61.2%)
Mean conditional estimate (95% CI) for the prevalence proportion^b^ where the 60^th^ percentile > threshold hours^c^	95.4% (88.8%–98.2%)	51.6% (34.6%–68.2%)

Table 4 presents the counts of all days, quarter and day-of-the-week combinations, and the number of facilities included in the analyses of Table 3, where at least two ORs were opened for cataract surgery. The total number of cataract surgeries and the pooled mean number of cataract surgeries per day are also shown.

**Table 4:** Number of lists corresponding to the analyses for the specified percentiles in Table 3. ^a^ The median (interquartile range) for the list duration was 5.21 (3.91–6.81) hours. ^b^ The median (interquartile range) for the list duration was 4.67 (3.53–6.46) hours. ^c^ The median (interquartile range) for the list duration was 6.61 (5.02, 7.42) hours.

Category	Number of lists corresponding to the prevalence proportions for the 66.7^th^ percentile^a^	Number of lists corresponding to the prevalence proportions for the 60^th^ percentile^b^	Number of lists corresponding to the prevalence proportions for the 80^th^ percentile^c^
Total days with cataract surgery	8,239	8,823	5,454
Quarter and day-of-the-week combinations	940	1086	506
Facilities	94	105	52
# of cataract surgeries	211,046	228,994	140,879
Mean cataract surgeries/day	25.6	26.0	25.8

Sensitivity analyses

The conditional estimates for the percentages of quarters and weekdays, corresponding to the estimate for the median facility, were even larger than the marginal estimates (Tables 1, 2). For facilities particularly adverse to overruns (i.e., a cost ratio of 4.0), the marginal estimate increased to 92.6%, whereas for a cost ratio of 1.5, the estimate remained substantive at 80.7% (Table 1). This sensitivity analysis indicates that the cost ratios do not affect our conclusions substantively. Sensitivity analyses for the marginal estimate for a threshold of 6.0 hours using the cost ratio of 2.0 indicate a smaller but still substantive percentage of facilities with lists exceeding this value (46.4%, 95% CI 42.7% to 50.2%, Table 1). There were very few facilities with lists exceeding 8.0 hours (Table 1), indicating that cataract surgery, overall, represents a relatively brief clinical workday. The corresponding sensitivity analyses among facilities opening at least two cataract surgery ORs are presented in Table 2.

Suppose more than 50% of cases performed with an anesthesia clinician could have been performed safely without their presence. Then, the shorter lists (Table 2) could be reduced, freeing up an anesthesia clinician. From the count of lists in Tables 1, 2, this would be ≥16% ($\tiny 100\times \frac{8239}{8329+42224}$). The primary result from Table 2 indicates that such a change would result in a reduction of at least one planned half-day session at most facilities. Potentially, the longer lists (Table 1) could be converted to half-day sessions with an anesthesia clinician; however, it is unlikely to be entirely removed based on the following arithmetic. For the cases analyzed in Table 1, the trimmed mean anesthesia time for each case was 27.2 minutes, and the turnover time was 7.1 minutes. Because we are assessing possible disruptions to care on the day of surgery, directly affecting the ophthalmologist, anesthesia clinician, and surgical nurses, we use the 80^th^ percentile (Table 1, last column). Twelve cases would be budgeted (with 11 turnovers), because $\tiny \frac{1.2\times 27.2+11\times 7.1}{60}$ = 6.74 hours, close to the 6.77 median list duration observed (Table 1). To have ≥80% of the lists completed successfully, the proportion of successive cases that would need to be completed successfully without needing an anesthesia clinician for any of the cases would need to be ≥98.2%, from the binomial distribution: $\tiny 0.982^{12}=0.80$. In other words, to entirely remove the care of anesthesia clinicians from the full-day sessions and to have a disruption no more often than once every five days, no more than 1.8% of patients could require the care of an anesthesia clinician.

## Discussion

The primary study result is that in the United States, nationwide, the session length to plan for the anesthesia clinician providing care during cataract surgery in the least busy OR (i.e., the fewest number of hours of surgery) exceeds 4.0 hours at the large majority (84.3%, Table 1) of quarters and weekdays. Furthermore, cataract surgery with anesthesia clinicians is not a low-caseload specialty [27], based on the high proportion of facility-workday combinations with at least one such list. One implication is that reducing the number of anesthesia clinicians (i.e., ORs) by one would substantially disrupt the cataract surgery workload for the large majority of lists with an anesthesia clinician. The 98.2% required proportion of patients in the list suitable for care without an anesthesia clinician to avoid disruption on 80% of lists is implausibly too large to achieve. That is, it should be expected that more than 1.8% of patients will have at least one comorbidity that requires them to be sedated and monitored by an anesthesia clinician for safety reasons. If the dates when patients have surgery were changed, with foreknowledge that an anesthesia clinician would be unavailable, it would reduce disruption to the surgical suite; however, this is not quantifiable from our available data. Furthermore, the feasibility from a patient perspective is not known. A previous study showed that the choice of day and morning/afternoon surgery was so important to older patients that they traveled more than one hour to a facility that provided such services [19].

Another implication is that a conceptual model in which most facilities performing cataract surgery with anesthesia clinicians have cataract surgery lists with anesthesia clinicians on one day of the week, but perhaps without anesthesia clinicians on other weekdays, is incorrect. Because of the large proportion of four-hour lists (i.e., typically comprising at least eight patients), even among facility-workdays with lists in multiple ORs (Table 2), there would be few opportunities to address case scheduling, such as consolidating cataract surgery performed several days of the week to a single day or changing from weekly to bi-weekly block time allocation [25]. The observation that many combinations of facility and day of the week exceed six hours of cataract surgery underscores this point. Even at facilities opening more than one OR for cataract surgery, the ability to decrease staffing by one anesthesia clinician or address case scheduling would be limited because of the substantive percentage (63.0%, Table 2) of four-hour lists. Thus, in the United States, ophthalmologists are filling their cataract surgery blocks with cases involving anesthesia practitioners. According to the findings from the Ophthalmology GIRFT Programme National Specialty Report, the same situation appears to exist in the UK [9].

Although many patients may be suitable candidates for cataract surgery without the involvement of an anesthesia clinician, there are some in whom the presence of an anesthesia clinician provides an increased margin of safety and an improved patient experience [8]. The probability of encountering patients needing the involvement of an anesthesia clinician in a long list is such that the ability to reduce anesthesia clinician staffing for the cataract surgery OR is limited. When the preoperative medical assessment of cataract surgical patients is performed on the day of surgery [4], some of the patients who require an anesthesia clinician are not identified in advance. Even when patients' medical records are available electronically for presurgical review, the extent of relevant physiological compromise may not be evident (e.g., whether a patient with congestive heart failure is able to tolerate lying flat), and psychological conditions such as trypanophobia or claustrophobia may not be articulated. Thus, not having any anesthesia clinicians assigned to provide sedation would likely disrupt the surgical workflow. As described by Duroi et al. in their consecutive review of 651 patients who all underwent successful cataract surgery under local eye drops, intracameral injection, and oral sedation, such disruptions can be mitigated where patients have in-person preoperative consultation before the day of cataract surgery to identify those with disqualifying conditions (e.g., anticipated lack of cooperation, intractable cough, tremor, cardiorespiratory conditions not allowing supine positioning, risk of vasovagal response, ocular pathology precluding care without an anesthesia clinician) [28]. Unfortunately, that report did not provide the percentage of screened patients who were suitable candidates [28]. Nevertheless, the percentage is an important consideration when assessing the potential impact of relocating cataract patients from ORs to procedural units for their care.

We emphasize that this is not an evaluation at the individual practice level, as insufficient information on patient comorbidities and other potentially disqualifying conditions was available in the database to determine the number that might be suitable to transfer intraoperative monitoring and sedation management to non-anesthesia clinicians. Such analysis would need to be done at the individual practice level to assess the potential for reducing the involvement of anesthesia clinicians during cataract surgery. What will likely become increasingly important is careful documentation in the preoperative evaluation of the medical necessity for anesthesia services during cataract surgery. For example, Anthem will deny coverage for cataract surgery unless an approved medical indication is documented [29]. Relatedly, the study does not provide insight into the potential reduction of such cataract anesthesia services at the national level based on the presence of such disqualifying factors. These are areas that likely require prospective study because psychological factors influencing a patient's suitability for cataract surgery without anesthesia practitioner care are often absent from administrative databases and seldom captured in electronic health records. Other areas that require investigation include large-scale assessments of access to cataract surgery in regions or countries where anesthesia care is not a covered service, sufficiently powered studies of outcomes in higher-risk patients, and surveys of patient preferences and their willingness to undergo cataract surgery in the absence of an anesthesia practitioner.

Limitations and strengths

A limitation of the study is that NACOR is a voluntary registry, not a random cross-section of United States anesthesia practices. However, it comprises many cases (n=16,335,242 records between 2022 and 2023) and includes many categories of anesthesia practices, from those working at small, community-based hospitals to large academic institutions. We are not aware of any factors that would make the care represented in the NACOR database atypical of anesthesia practices in the United States. The fact that data from NACOR are being used by the ASA for national performance benchmarking and quality reporting should provide some reassurance that the results from this study are generalizable [14]. The 754,718 cataract cases in the NACOR database over the two-year study interval represent approximately 10% of the estimated 3.7 million cataracts performed annually in the United States during the interval covered by the analyzed dataset [1]. Another limitation is that there were insufficient patient data to assess the fraction of these patients who underwent cataract surgery with care provided by an anesthesia clinician, potentially suitable candidates for local anesthesia in the absence of an anesthesia clinician. Similarly, because only cases involving an anesthesia clinician are reported to the registry, we cannot determine the percentage of cataract surgery being performed without an anesthesia clinician's involvement. A potential limitation is that more than one anesthesia group was present at a facility providing care for patients undergoing cataract surgery, but data were not reported to NACOR for all groups. Although it is not expected that this would be a common situation for cataract surgery, the effect would be that our estimates of the length of the day and the number of cases performed would be underestimated, thereby strengthening our conclusions. Finally, the data are from a single country (the United States) and may not reflect practices in other countries.

## Conclusions

The durations of the shortest cataract surgery lists in the United States with an anesthesia clinician are sufficiently long that suitable session lengths would exceed 4.0 hours for the large majority of facilities where cataract surgery is regularly performed with an anesthesia clinician. Most (71.3%) such facilities have only one list (i.e., one OR) daily. The implication is that a reduction of anesthesia staffing by even one anesthesia clinician (i.e., one staffed OR) would likely result in a substantive disruption in the cataract surgery workload, given the number of patients receiving care and the probability that some of the patients will require the presence of an anesthesia clinician to manage their intraoperative sedation and medical care. Given this requirement, potential policy decisions by insurance companies or governmental agencies to remove the provision of anesthesia care as a covered healthcare service would be unjustified. Facilities should not plan to have zero anesthesia clinicians caring for cataract patients on a daily basis. Rather, the results suggest an equilibrium of one clinician (or room) per day with such a list.

## References

[1] Rossi T, Romano MR, Iannetta D, Romano V, Gualdi L, D'Agostino I, Ripandelli G (2021). Cataract surgery practice patterns worldwide: a survey. BMJ Open Ophthalmol.

[2] Clare G (2019). Cochrane corner: patient safety in cataract surgery. Eye (Lond).

[3] Perumal D, Dudley RA, Gan S (2022). Anesthesia care for cataract surgery in medicare beneficiaries. JAMA Intern Med.

[4] Keay L, Lindsley K, Tielsch J, Katz J, Schein O (2019). Routine preoperative medical testing for cataract surgery. Cochrane Database Syst Rev.

[5] Neo YN, Gruszka-Goh MH, Braga AJ (2023). Royal College of Ophthalmologists' National Ophthalmology Database study of cataract surgery: report 11, techniques and complications of local anesthesia for cataract surgery in the United Kingdom. J Cataract Refract Surg.

[6] Kugler LJ, Kapeles MJ, Durrie DS (2023). Safety of office-based lens surgery: U.S. multicenter study. J Cataract Refract Surg.

[7] Whelan H, Malcolm J, Buchan JC (2023). Comment on: safety of office-based lens surgery: a U.S. multicenter study. J Cataract Refract Surg.

[8] Palte HD, Gayer S, Kumar C (2010). Role of the anaesthetist during cataract surgery under local anaesthesia. Br J Anaesth.

[9] National Health Service (2025). Ophthalmology: GIRFT Programme National Specialty Report. https://gettingitrightfirsttime.co.uk/wp-content/uploads/2019/12/OphthalmologyReportGIRFT19S.pdf.

[10] Holmer H, Lantz A, Kunjumen T (2015). Global distribution of surgeons, anesthesiologists, and obstetricians. Lancet Glob Health.

[11] Abouleish AE, Pomerantz P, Peterson MD (2024). Closing the chasm: understanding and addressing the anesthesia workforce supply and demand imbalance. Anesthesiology.

[12] National Health Service (2025). Anaesthesia in Cataract Hubs. https://www.gettingitrightfirsttime.co.uk/wp-content/uploads/2020/12/Anaesthesia-in-Cataract-Hubs-FINAL.pdf.

[13] Molina L, Lyang N, Schwartz R, Parikh N, Ramanathan S, Huang AJ, Chen CL (2025). Patient perspectives and concerns regarding cataract surgery and cataract surgery sedation: a qualitative study. Clin Ophthalmol.

[14] (2025). National Anesthesia Clinical Outcomes Registry (NACOR). https://www.asahq.org/aqi/registries/nacor.

[15] Epstein RH, Dexter F, Fahy BG (2024). Since the COVID-19 pandemic, approximately 90% of elective anesthetics have been ambulatory: a retrospective analysis of statewide data in Florida from 2010 through 2022. J Clin Anesth.

[16] McIntosh C, Dexter F, Epstein RH (2006). The impact of service-specific staffing, case scheduling, turnovers, and first-case starts on anesthesia group and operating room productivity: a tutorial using data from an Australian hospital. Anesth Analg.

[17] Dexter F, Dutton RP, Kordylewski H, Epstein RH (2015). Anesthesia workload nationally during regular workdays and weekends. Anesth Analg.

[18] Dexter F, Epstein RH, Rodriguez LI (2019). Throughout the United States, pediatric patients undergoing ambulatory surgery enter the operating room and are discharged earlier in the day than are adults. Periop Care Oper Room Manag.

[19] Dexter F, Birchansky L, Bernstein JM, Wachtel RE (2009). Case scheduling preferences of one surgeon's cataract surgery patients. Anesth Analg.

[20] (2025). American Medical Association. CPT® overview and code approval. https://www.ama-assn.org/practice-management/cpt/cpt-overview-and-code-approval.

[21] (2025). Clinical classifications software (CCS) for ICD-9-CM. https://hcup-us.ahrq.gov/toolssoftware/ccs/ccs.jsp.

[22] Strum DP, Vargas LG, May JH, Bashein G (1997). Surgical suite utilization and capacity planning: a minimal cost analysis model. J Med Syst.

[23] Wachtel RE, Dexter F (2010). Review article: review of behavioral operations experimental studies of newsvendor problems for operating room management. Anesth Analg.

[24] Wachtel RE, Dexter F (2007). A simple method for deciding when patients should be ready on the day of surgery without procedure-specific data. Anesth Analg.

[25] Hyndman RJ, Fan Y (1996). Sample quantiles in statistical packages. Am Stat.

[26] Dexter F, Epstein RH (2006). Holiday and weekend operating room on-call staffing requirements. Anesth Analg.

[27] Dexter F, Epstein RH, Podgorski EM 3rd, Pearson AC (2020). Appropriate operating room time allocations and half-day block time for low caseload proceduralists, including anesthesiologist pain medicine physicians in the State of Florida. J Clin Anesth.

[28] Duroi Q, Baudet JM, Bigoteau M, Slim M, Pichard T, Pisella PJ, Khanna RK (2021). Author correction: ambulatory cataract surgery centre without perioperative anesthesia care: a prospective cohort study. Sci Rep.

[29] (2025). Monitored anesthesia care and MAC for cataract surgery. https://www.asahq.org/~/media/sites/asahq/files/public/advocacy/alerts/washington%20alerts/2018-02-28-cg-med-60-monitored-anesthesia-care-and-general-anesthesia-for-cataract-surgery.pdf?la=en.

